# Social Media Programs for Outreach and Recruitment Supporting Aging and Alzheimer Disease and Related Dementias Research: Longitudinal Descriptive Study

**DOI:** 10.2196/51520

**Published:** 2024-07-09

**Authors:** Anthony L Teano, Ashley Scott, Cassandra Gipson, Marilyn Albert, Corinne Pettigrew

**Affiliations:** 1 Department of Geriatric Medicine and Gerontology Johns Hopkins University School of Medicine Baltimore, MD United States; 2 Department of Neurology Johns Hopkins University School of Medicine Baltimore, MD United States

**Keywords:** education, social media, outreach, recruitment, Alzheimer’s disease, Alzheimer disease

## Abstract

**Background:**

Social media may be a useful method for research centers to deliver health messages, increase their visibility in the local community, and recruit study participants. Sharing examples of social media–based community outreach and educational programs, and evaluating their outcomes in this setting, is important for understanding whether these efforts have a measurable impact.

**Objective:**

The aim of this study is to describe one center’s social media activities for community education on topics related to aging, memory loss, and Alzheimer disease and related dementias, and provide metrics related to recruitment into clinical research studies.

**Methods:**

Several social media platforms were used, including Facebook, X (formerly Twitter), and YouTube. Objective assessments quantified monthly, based on each platform’s native dashboard, included the number of followers, number of posts, post reach and engagement, post impressions, and video views. The number of participants volunteering for research during this period was additionally tracked using a secure database. Educational material posted to social media most frequently included content developed by center staff, content from partner organizations, and news articles or resources featuring center researchers. Multiple educational programs were developed, including social media series, web-based talks, Twitter chats, and webinars. In more recent years, Facebook content was occasionally boosted to increase visibility in the local geographical region.

**Results:**

Up to 4 years of page metrics demonstrated continuing growth in reaching social media audiences, as indicated by increases over time in the numbers of likes or followers on Facebook and X/Twitter and views of YouTube videos (growth trajectories). While Facebook reach and X/Twitter impression rates were reasonable, Facebook engagement rates were more modest. Months that included boosted Facebook posts resulted in a greater change in page followers and page likes, and higher reach and engagement rates (all *P*≤.002). Recruitment of participants into center-affiliated research studies increased during this time frame, particularly in response to boosted Facebook posts.

**Conclusions:**

These data demonstrate that social media activities can provide meaningful community educational opportunities focused on Alzheimer disease and related dementias and have a measurable impact on the recruitment of participants into research studies. Additionally, this study highlights the importance of tracking outreach program outcomes for evaluating return on investment.

## Introduction

With the aging of the population, the number of individuals living with dementia globally is expected to triple by 2050 [1]. Disseminating reliable information on topics related to healthy aging and Alzheimer disease and related dementias (ADRD) is particularly important given modifiable lifestyle factors may play a role in dementia risk reduction [2], receiving an early diagnosis requires identifying the signs and symptoms of memory loss, and persons and families affected by dementia may benefit from information about ADRD. Connecting the public with education and resources related to these topics is aligned with national and international initiatives that emphasize the importance of healthy aging, dementia awareness, risk reduction, diagnosis and services, and caregiver support [3,4]. Social media content aimed at older adults may provide a mechanism for sharing resources, research findings, and available services related to healthy aging and ADRD. Social media may also generate increased interest in research participation, in addition to the in-person approaches that have been used for many years.

Research centers specializing in aging and ADRD use several approaches for community outreach, education, and volunteer recruitment for research studies. This includes a variety of in-person events, such as providing educational presentations in the community, hosting resource tables at community events, and organizing conferences in collaboration with community organizations, among others. Centers also use a range of web-based activities including newsletters, websites, and social media platforms such as Facebook, X (formerly Twitter), YouTube, and Instagram. While social media may be a potentially important method of delivering health messaging and recruitment [5], the types of activities used, and the outcomes of social media outreach efforts, are not well understood.

Social media has become a primary source of information for the general public hoping to “seek and share health-related information” [6]. Recent estimates indicate that 73% of middle-aged and 45% of older adults in the United States use social media, with Facebook and YouTube being two of the most common among these age groups [7,8]. This suggests that key age demographics for messaging may be reached through social media, providing a platform for research centers to become trusted web-based sources of information on topics related to healthy aging and memory loss, ADRD, and caregiving in their local communities.

Studies incorporating social media activities into community outreach and recruitment in the context of aging and memory loss have covered a broad range of topics. For example, studies have examined the role of social conversations in providing advice related to cognitive decline [9]; described social media activities in targeted dementia awareness campaigns [10-12]; and evaluated the use of social media advertisements to drive traffic to educational resources [13] or to recruit into aging or ADRD research studies [14-16]. Moreover, prior research has demonstrated the feasibility of using a digital community-engaged research approach (which included a social media component) for reaching individuals from racially diverse backgrounds [17]. More broadly, studies analyzing dementia content on social media have also suggested that these platforms have the potential to deliver health information on this topic, raising awareness and facilitating communication with the public [18-20].

Prior studies in this area have not, to our knowledge, described aging and memory loss educational programs across multiple social media platforms and reported on their impact on research recruitment. To address this gap, this study describes the social media approaches, aimed at community outreach and education related to aging and ADRD, implemented by one research center. Importantly, the objective was not to compare social media to other approaches but to describe the social media activities implemented to date and evaluate the outcomes of these activities using up to 4 years of metrics, including success in reaching members of the community and impact on recruitment.

## Methods

### Overview

The Johns Hopkins Alzheimer’s Disease Research Center (JHADRC) has used 3 social media platforms over the past 4 years: Facebook, Twitter (rebranded as X in 2023), and YouTube. Each platform was launched with a different set of goals.

#### Facebook

The primary goal of the *Johns Hopkins Memory & Aging* Facebook page is to provide education and resources, as well as information about community events and research opportunities, related to healthy aging, brain health, memory loss, and dementia. The content primarily targets members of the community and community partners. Content is posted to this page 3-5 times per week. This page was launched in January 2019.

#### Twitter

The goals of the *Johns Hopkins Memory & Aging* Twitter account are 3-fold. The first goal is consistent with those of the Facebook page: to provide education, resources, and information about community events and research opportunities related to healthy aging, brain health, memory loss, and dementia. This includes promoting the visibility of Johns Hopkins (JH) aging and ADRD researchers, clinicians, and staff. The second goal is to serve as an information hub for local aging and ADRD researchers by sharing academically focused content such as recent research publications, information about social determinants of health for underserved populations (eg, Black Americans, Hispanics, and the LGBTQ+ community), funding and training opportunities, professional development opportunities, and upcoming conferences. Where possible, content is shared (ie, retweeted) through related JH Twitter accounts, such as the Center on Aging and Health and Geriatric Medicine and Gerontology, to expand views of the content. The third goal is to strategically network with community partners engaged in activities related to aging and ADRD. This includes making a concerted effort to share the events, activities, and accomplishments of these groups. Given these 3 broad goals, the target audience includes community audiences and community partners, as well as researchers and health professionals. A communications specialist spends approximately 1-3 hours per weekday identifying, sharing, and retweeting content of relevance to the abovementioned audiences, and approximately 6-8 hours monthly creating original content. This account was launched in July 2020.

Readers should note that the Facebook and Twitter pages are branded with the name *Johns Hopkins Memory & Aging*. This name was selected to be intentionally broad in order to demonstrate relevancy to topics related to aging, memory loss, and ADRD, and to resonate with the general public, particularly the target audience of middle-aged and older adults.

#### YouTube

The primary goal of the *Johns Hopkins ADRC* YouTube page is to serve as a repository for outreach content, including center-produced resources, recordings of web-based community outreach events, and activities developed in collaboration with the JHADRC’s community advisory board, known as the Memory and Aging Community Advisory Board (MACAB). The page primarily targets members of the community, with content uploaded as needed. This page was launched in October 2020.

All social media pages are overseen by members of the JHADRC’s Outreach, Recruitment, and Engagement (ORE) Core. The ORE Core worked with the Johns Hopkins Medicine Strategic Marketing and Outreach team to set up the page and get approval for the use of the institutional name. The web-based recruitment materials of the JHADRC were approved by the Johns Hopkins Medicine institutional review board (IRB).

### Description of Content

The majority of content shared through the Facebook and Twitter pages highlights topics broadly related to ADRD, consistent with the goals of these pages. This includes educational content related to healthy aging, brain health, and risk factors for memory loss; information about Alzheimer disease and other types of dementia; resources for caregivers; information about clinical research and research findings; social determinants of health; and local events of relevance to these topics (both our own and those of our community partners). During the first year of the COVID-19 pandemic, many posts included COVID-19–related health and support resources.

The shared information includes content developed by JH staff (described below) and content from external sources such as news articles, website pages, infographics, and blog posts from trusted outlets. For example, we frequently share content from the National Institute on Aging, the Alzheimer’s Association, our local Alzheimer’s Association Chapter, the American Association of Retired Persons (AARP), a website known as MindCrowd, and major news outlets. We also make a concerted effort to share news articles and web-based resources that quote or feature JH staff, researchers, or clinicians, as well as materials that highlight recent research findings from studies that include JH researchers. As noted above, our Twitter page also shares content of relevance to academic audiences and health professionals, such as funding and conference announcements and professional development opportunities.

### Internally Developed Social Media Content and Programs

#### Social Media Series

To date, we have developed 3 educational series consisting of a collection of posts on specific topics, including general information about AD, brain health, and research awareness (see Table 1 for details). Posts within each series are branded with a hashtag, allowing the topic to be indexed and searchable on social media platforms. Although developing a series of posts requires time up-front, the approach provides a library of original content that can be released over several months or more, and are available to be reposted in the future. This also allows for content to undergo IRB review prior to use if needed (eg, Research Awareness Series). Having prepared content on-hand has been particularly helpful given the multiple responsibilities that compete for staff effort.

**Table 1:** Overview of social media series about aging and ADRD^a^.

Series name (social media hashtag)	Goal of content and distribution timeline	Description of content	Posts, n
Alzheimer’s awareness series (#JHAlzAwareness)	Increase awareness of AD^b^ Developed and posted in 2020; some content reposted 2021 and 2022	Two categories of content: (1) education about AD (eg, definitions, prevalence, signs and symptoms, stages, effect on the brain, and importance of research) and (2) risk factors and symptom management (eg, risk factors, risk reduction, genetics, diagnosis, treatments, and caregiving)	31
Brain health series (#JHBrainHealthMatters)	Share expert recommendations and practical tips from the reports developed by the AARP’s^c^ Global Council on Brain Health Developed and posted in 2020; plans to repost some content in future	Content and infographics from Global Council on Brain Health reports: (1) The Brain-Sleep Connection; (2) The Brain-Body Connection; (3) Engage Your Brain; (4) Music on Our Minds; (5) The Brain-Heart Connection; (6) Brain Food; (7) The Real Deal on Brain Health Supplements; (8) Preserving Your Brain Health During Illness or Surgery; (9) Brain Health and Mental Well-Being; and (10) The Brain and Social Connectedness	89 (7-10 posts per topic)
Research awareness series (#JHMemoryResearch)	Increase awareness of the importance of memory loss research and ongoing research activities to facilitate recruiting participants into JHADRC^d^-affiliated research studies Developed in 2021, posted 2022-2023	Topics covered: (1) goals of research and different types of research studies (observational, interventions, or clinical trials); (2) examples of research procedures and what’s learned (eg, brain imaging and fluid biomarkers); (3) importance of diversity in research; (4) examples of JHADRC-affiliated research studies; (5) benefits of research participation; (6) participant, staff, and researcher spotlights; (7) study recruitment flyers	86

^a^ADRD: Alzheimer disease and related dementias.

^b^AD: Alzheimer disease.

^c^AARP: American Association of Retired Persons.

^d^JHADRC: Johns Hopkins Alzheimer’s Disease Research Center.

#### Web-Based Talks About Memory Loss and Brain Health on Social Media

We have developed brief web-based presentations designed to reach new audiences in the local community. Web-based presentations on the topics of memory loss (“What you should know about memory loss”) or brain health (“A brain healthy lifestyle matters for healthy aging”) are given by ORE Core Community Outreach Coordinators. Individuals register through Zoom and registrants must attend to receive the content (ie, presentations are not recorded). One week prior to the presentation date, modest funds (US $30-US $150) are used to boost the post on Facebook, targeting middle-aged and older adults in the Greater Baltimore area. These presentations, organized approximately every quarter, have been advertised 11 times to date, at various times of day (eg, noon “lunch & learn”; evening “dinner table discussion”). Typically, more individuals register than actually attend. On average, 19 individuals, representing approximately 25% of those registered, attend each presentation.

#### Educational Videos Posted to YouTube

A web-based talk series, titled *Memory Matters*, was developed to provide brief research updates on topics related to aging, memory loss, and ADRD. These approximately 15-minute presentations are designed to share recent research findings that may be of particular interest to lay audiences, such as sleep, hearing loss, and physical activity (see the “ Growth Trajectories and Page Visibility” section for details). To ensure that the presentations are targeted to a lay audience, presenters are asked to include an overview slide, make their slide content as simple and clear as possible, include a summary slide that recaps main points, conclude with actionable takeaway messages, and share publicly available resources. They also receive a document entitled “Tips and Examples for Effective Science Communication to a Lay Audience.” Presenters are given a template for the first and last slide to provide uniformity to the talks within the series. To date, these talks have been given by junior faculty who are given feedback in advance of the video recording by center faculty (CP and MA). Thus, this talk series also provides an opportunity for science communication mentorship to junior investigators, including guidance on effectively communicating complex scientific topics with lay audiences. Talks within this series are branded with the hashtag #JHMemoryMatters. Once recorded and edited, they are posted to YouTube and the JHADRC website and shared through Facebook and Twitter.

A *Community Views* (#CommunityViews) web-based interview series features short (6-7 minutes) one-on-one interviews with members of the community. The goal of these videos is to provide members of the lay community an opportunity to advocate for topics of personal importance to them. Members of the JHADRC ORE Core work with the interviewee to collaboratively develop a set of interview questions, as well as a short set of slides to support the interview content and provide uniformity to the talks within the series. Once recorded and edited, the interviews are posted on YouTube and shared through Facebook and Twitter. To date, the interviews have been with members of the MACAB. Two interviews have been recorded: the first on the importance of educating the youth about dementia and the second on empowering older adults and caregivers to maintain their brain through physical activity and self-care. These programs are ongoing.

#### Twitter Chats and Webinars

A series of Twitter chats and webinars have been developed to promote the goals of a coalition of stakeholders in the brain health and dementia community. This collaboration consists of 2 research centers internal to Johns Hopkins University (the JHADRC and the Johns Hopkins Alzheimer’s Disease Resource Center for Minority Aging Research), as well as external partners including the Global Council on Brain Health convened by the American Association of Retired Persons, the Greater Maryland Chapter of the Alzheimer’s Association, and 2 strategically targeted audience stakeholder organizations, both local alumnae chapters of the Delta Sigma Theta Sorority, Inc. Branded as #BrainMatters, the goal has been to develop dialog, engagement, and educational programs for sharing evidence-based information about brain health, health disparities, memory loss, and ADRD at the grassroots level. This approach aligns with the recognized importance of developing equitable community partnerships for reaching individuals from diverse backgrounds [5].

Prior to each scheduled event, the program organizers communicate via email and meet virtually (typically 3-5 times) to make collaborative decisions about program timing, format, content, and invited participants. For Twitter chats, this involves identifying the specific questions to be sequentially asked during the scheduled web-based conversation; questions are answered by both invited guests and the Twitter users at large. For webinars, this involves deciding on the topic and event flow (eg, speaker presentations vs moderated conversations). To date, Twitter chat topics have included (1) brain health, (2) achieving brain health equity, and (3) the state of research on memory loss and dementia, and webinar topics have included (4) dementia caregiving and caregivers and (5) the relationship between community, social connection, and cognitive decline. Event promotion occurs predominantly on Twitter (which allows us to tag guest speakers) and Facebook, and is shared by the coalition’s stakeholders. The product of the Twitter chats remains on Twitter indefinitely as a resource to the public, and webinars have been recorded and uploaded to YouTube, thus, generating a marketable product that can also be shared after the event. In 2023, the #BrainMatters leadership made the decision to pivot toward webinars over Twitter chats, as it was determined that this format would better reach the target audiences.

### Overarching Strategies

#### Research Recruitment

When the Facebook and Twitter accounts were first launched, we strategically decided not to emphasize clinical research recruitment (eg, study flyers, information about ongoing studies). Our goal was to first build a modest base of followers and develop a presence as a source of information before discussing research participation. Information about ongoing clinical research studies was not consistently posted until approximately 3 years after the Facebook page was launched. To date, this has been accomplished primarily through our Research Awareness Series (described above and in Table 1).

#### Diversity and Inclusion

We take care to ensure the visuals we post are representative of individuals from diverse communities. This includes evaluating the pictures posted in association with existing website links, as well as making a concerted effort to ensure that stock photos reflect individuals from diverse racial and ethnic backgrounds.

#### Boosting Facebook Posts

We have boosted occasional Facebook posts to increase the reach of our content and enhance the local visibility of our efforts, our Facebook page, and our center more broadly. This involves paying Facebook to more prominently display specific posts in users’ feeds. To help reach new local audiences who may be interested in our content, boosted posts targeted middle-aged and older adults (eg, 55 years and older) in the Greater Baltimore area (ie, approximately 25- to 50-mile radius of Baltimore, MD) using the Age and Locations fields of Meta Business Suite’s Boost Post settings. This strategy relied on Meta’s “Audience” settings, which allow users to define who will see their advertised posts by targeting audiences whose profiles match specific characteristics, such as demographics. Each time content is boosted, we make a concerted effort to invite individuals who like the posts to like or follow our Facebook page, to help expand our followership. Boosted content has most frequently included talks on social media, as well as posts from our educational series (eg, the Research Awareness Series) and information about center-organized events (eg, the MACAB’s Annual Holistic Health Seminar on Memory Loss). To date, our expenditures have been modest, ranging from US $30 to US $200 per boosted post. In years 2 and 3, we boosted 1 post approximately every other month; from year 4 onwards, we boosted 1 post almost every month.

### Measures and Outcomes

In order to track our social media activities, at the start of each new month, members of the ORE Core record metrics from the prior month using data from each platform’s native analytics dashboards.

For Facebook, the following metrics were tracked through the Meta Business Suite’s Professional Dashboard: number of posts made during the month; the average reach of that month’s posts, reflecting the number of people who saw that month’s post at least once (calculated as the total [sum] reach of all posts within the month divided by the total number of posts); and the average engagement with that month’s posts, reflecting the number of times people engaged with that month’s posts through reactions, comments, shares, and clicks (calculated as the total [sum] engagement with that month’s posts divided by the total number of posts). Note that all posts within a given month were included in these monthly analytics; therefore, the data reflect both organic and paid reach and engagement. The number of page likes and page followers was additionally recorded. Facebook metrics were missing for 2 months in year 2.

For Twitter, the following metrics were tracked through Twitter’s monthly Analytics report: number of Tweets; Tweet impressions, reflecting the number of times that month’s Tweets were displayed to users; profile visits; mentions; and new followers. The number of account followers and the number of accounts that we follow were additionally recorded. Two Twitter metrics (the number of account followers and the number of accounts we follow) were missing for 5 months in year 1, 2 months in year 2, and 2 months in year 3.

In this report, we summarize metrics for both Facebook and Twitter. This includes monthly data on the number of likes and followers to assess page growth trajectories (presented in 6-month intervals), which has previously been described as an indicator of success [5]. Monthly metrics were additionally used to calculate average reach rates and average engagement rates for Facebook, and average impression rates for Twitter. These were calculated by dividing each month’s average reach, average engagement, or average impressions by that month’s total number of page followers, then multiplying the quotient by 100. Annual reach, engagement, and impression rates were calculated for all years (excluding year 1) by averaging over all available months within a year, for year 2 onwards. Year 1 metrics were excluded because they appear inflated due to high engagement from a limited number of followers, as the pages were being established and accruing audiences. Note that the analytics for posts made at the end of the month may be slightly underestimated, given posts may have received continued views after the data were recorded. For videos posted to YouTube, we report the number of views over time.

The number of individuals who expressed interest in participating in research after engaging with our social media activities was tracked through a secure, web-based REDCap database hosted at Johns Hopkins [21,22]. This database is designed to track the outreach activities that result in recruitment, including how an individual learned about our studies, whether they were referred to a center-affiliated research study, and the outcomes of those referrals. Database entries that provide results related to social media come from 2 sources of information. The first reflects individuals who complete an IRB-approved “Participate in Research” webform on the JHADRC website and indicate on that form that they learned about us through social media. The second pertains to entries of individuals who contact us (eg, by phone) to express interest in research after viewing web-based programming, indicating that they learned about our programs through social media while completing their phone screen; these individuals were entered into the database by members of the ORE Core.

### Statistical Analysis

The data reported below reflect outcomes and metrics between page launch (as noted by the dates above) and the time of manuscript writing (April 2023). Time-based outcomes (such as growth trajectories and video views) are reported as months since launch, to describe, for example, how long the content or programs have been available. Where relevant, continuous variables are summarized as mean (SD) and categorical data are summarized as n (%). Average annual reach, engagement, and impression rates are calculated as percentages (as described above), with differences between months with versus without boosted posts assessed by Mann-Whitney *U* tests. Analyses were run in SPSS (IBM Corp; version 29.0).

Social media data pertaining to this report are available by qualified investigators upon request to the corresponding author.

### Ethical Considerations

The data for this report come from 2 recruitment procedures implemented by the JHADRC, both of which have been approved by the Johns Hopkins Medicine IRB (protocol NA_00045104). First, the JHADRC website includes a section labeled “Participate in Research.” This section includes a web form that asks individuals interested in learning more about research to complete an electronic form. The first item on the webform asks that the individual respond “yes” or “no” to the following statement: “I understand that by submitting this form I am providing consent to be contacted about research studies related to aging and memory loss by staff affiliated with the Johns Hopkins Alzheimer's Disease Research Center, and that this information will be stored in a secure database.” Second, members of the JHADRC also administer an IRB-approved phone screen, which includes questions similar to those in the web form. The first item on the phone screen includes the following statement “Before we begin, I should point out that the possible risk to your answering questions is that you will be revealing confidential information to us. We promise to keep all such information strictly confidential. You do not have to answer any questions that might make you uncomfortable. Your participation is voluntary. If you do not agree, this will not affect your care, if you are a patient at Johns Hopkins, in any way. If you are not interested, please let me know.” The anonymized, group-level demographic data included in this study are based only on outcomes from individuals who responded “yes” to the statement at the beginning of the web form or completed the phone screen and indicated willingness to be contacted about research. Participants were not compensated for completing these optional forms and outcome data are reported in aggregate to protect participant privacy and confidentiality.

## Results

### Outcomes and Indicators of Success

#### Growth Trajectories and Page Visibility

**Figure 1:**
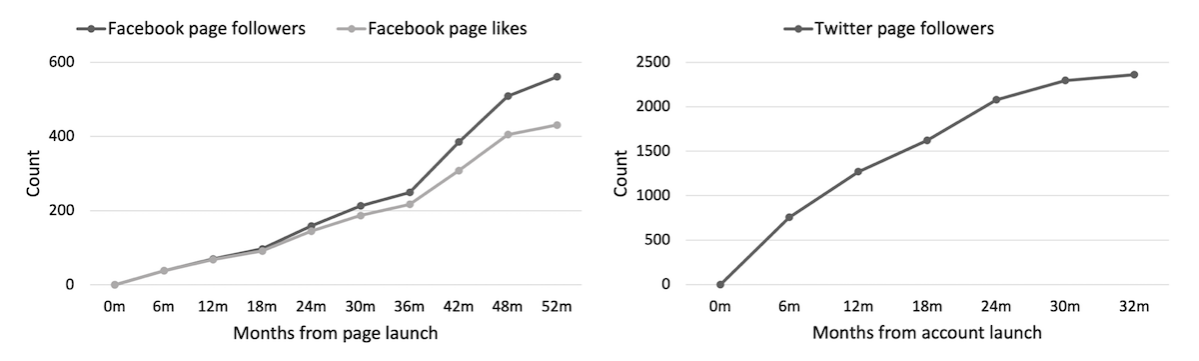
Growth trajectories in social media followers for Facebook (left; launched January 2019) and Twitter (right; launched July 2020), shown in 6-month intervals.

Growth trajectories for our Facebook and Twitter pages are shown in Figure 1. Both accounts have demonstrated continued increases in the number of new followers since the account launch, suggesting that it takes years to build a modest audience. The total number of Facebook followers exceeds 500; this includes a clear uptick in the trajectory of Facebook page followers around 24 months when we started boosting occasional Facebook posts. Notably, months that include a boosted Facebook post resulted in a greater change in page followers and page likes (mean change in page followers 13.7, SD 9.2; mean change in page likes 19.0, SD 12.3), compared with months that did not include boosted posts (mean change in page followers 6.2, SD 4.5, *U*=294.5, *P*<.001; mean change in page likes 8.0, SD 5.0, *U*=285.0, *P*=.002), suggesting our efforts to invite individuals to like or follow the page were successful. The total number of Twitter followers exceeds 2000. Additionally, the talks within the *Memory Matters* web-based talk series have received over 3000 views, whereas views of the *Community Views* interviews have been less (Table 2); both of the #BrainMatters webinars hosted to date attracted >100 registrants with >50 individuals attending.

Average annual reach and engagement rates (Facebook) and average annual impression rates (Twitter) for year 2 onward are shown in Table 3. As anticipated, for Facebook, average reach and engagement rates were significantly higher for months that included boosted posts (mean reach rate 136.2%, SD 87%; mean engagement rate 10.7%, SD 6.1%) compared with months that did not include boosted posts (mean reach rate 34.7%, SD 26%, *U*=332.0, *P*<.001; mean engagement rate 5.1%, SD 4.3%, *U*=297.0, *P*<.001).

**Table 2:** Overview of educational videos posted to YouTube, including Memory Matters web-based talk series (#JHMemoryMatters) and Community Views (#CommunityViews) interview series.

Talk title		Months since launch, n	Views to date, n
***Memory Matters*** **web-based talk series**			
	Dementia and cognitive decline: a brief overview	42	658
	Vascular risk factors for cognitive decline and dementia	41	430
	Changes in sleep and circadian rhythms in aging and memory loss	22	414
	Hearing loss and dementia: what’s the connection?	17	1629
	The importance of brain donation for Alzheimer’s disease and related disorders	5	90
	Physical activity and cognitive health	1	74
***Community Views*** **interview series**			
	Educating the youth about dementia	24	95
	Empowering older adults and caregivers	3	35

**Table 3:** Visibility metrics over time for Facebook and Twitter. Values reflect mean (SD) and ranges.

Year^a^	Facebook				Twitter		
	Average annual reach rate		Average annual engagement rate		Average annual impression rate		
	Mean (SD), %	Range	Mean (SD), %	Range	Mean (SD), %	Range	
All years (except year 1)	80.1 (79.1)	8.0-355.3	7.6 (5.8)	1.2-29.2	1260.3 (1076)	559.4-4152.9	
Year 2	50.2 (30)	26.7-113.9	8.2 (4.7)	4.1-16.5	1564.9 (1319.6)	559.4-4152.9	
Year 3	52.5 (37.7)	13.5-128.9	5.4 (2.7)	2.1-11.2	803.4 (195.6)	573.5-1141.6	
Year 4	138.4 (111.4)	11.6-355.3	10 (8.3)	1.4-29.2	—^b^	—	
Year 5	62.8 (61)	8.0-122.5	5.7 (5.3)	1.2-12.0	—	—	

^a^Metrics exclude each platform’s first year of data because these metrics appear inflated due to high engagement from a limited number of followers, as the pages were being established and accruing followers. For Facebook (launched in January 2019), year 2: January 2020-December 2020; year 3: January 2021-December 2021; year 4: January 2022-December 2022; year 5 (partial, n=4 months): January 2023-April 2023. For Twitter (launched in July 2020), year 2: August 2021-July 2021; year 3 (partial, n=8 months): August 2022-March 2023.

^b^Not available.

#### Research Recruitment Resulting From Social Media Activities

Social media activities have resulted in 89 individuals expressing interest in participating in center-affiliated clinical research studies over the past 2 years, the majority coinciding with boosted social media content (eg, posts from the #JHMemoryResearch series or web-based talks advertised on social media). This includes 76 individuals who indicated that they came to the JHADRC website after viewing social media content, when completing the “Participate in Research” website form (mean age 66.2, SD 10.4 years; 67/76, 88% female; 12/76, 16% self-reporting non-White race or Hispanic/Latino ethnicity). Of these, 46 (61%) have been referred to an ongoing research study, 22 (29%) were not referred, and 8 (11%) have phone screens pending (see Table 4 for additional details). In addition, 13 individuals expressed interest in participating in research after attending a web-based talk advertised on social media (mean age 64.3, SD 4.4 years; 13/13, 100% female; 3/13, 23% self-reporting non-White race). Of these, 12 (92%) have been referred to an ongoing research study; 1 (8%) was not referred (Table 4).

**Table 4:** Recruitment outcomes: breakdown of participant referrals for those expressing interest in participating in research after learning about the Johns Hopkins Alzheimer’s Disease Research Center through social media activities.

		Webform indicates social media as a source for engagement (n=76), n (%)	Attended a social media-advertised web-based talk (n=13), n (%)
**Referred to a center-affiliated research study**			
	Enrolled in a center-affiliated research study	6 (8)	5 (38)
	Enrollment pending (referral sent, outcome pending)	26 (34)	6 (46)
	Not enrolled (eg, ineligible; study coordinator unable to contact)	9 (12)	1 (8)
	Referred to another ADRC or online registries (eg, lives out of state; unable to travel)	5 (7)	0 (0)
**Not referred to a center-affiliated research study**			
	Phone screen pending	8 (11)	0 (0)
	Contacted but no longer interested	3 (4)	1 (8)
	Unable to contact	19 (25)	0 (0)

## Discussion

### Principal Findings

This study describes one center’s social media strategy for community outreach and recruitment. Four years of page metrics and recruitment data suggest that the content and programs provide educational resources, increase the visibility of the center’s activities, and result in the recruitment of participants into center-affiliated research studies.

Given the increased use of social media among middle-aged and older adults, these platforms can serve as one method by which the JHADRC provides reliable education and resources to members of the local community, networks (on the web) with local community organizations, and shares information about the important work (research studies, research findings, events, etc) being done by center-affiliated faculty and staff. Based on these data, it appears that these efforts, including boosting occasional Facebook posts, have increased the visibility of the JHADRC in the local geographic area and have provided opportunities to reach individuals who we may not have encountered through in-person community outreach. Importantly, these activities can be achieved primarily through staff time, with minimal additional cost to center budgets. Using creative digital approaches to serve as a public source of reliable information on topics related to aging, memory loss, and ADRD provides an additional opportunity for centers to meet critical community engagement goals, including building trust and creating better communication with members of the community [23,24].

These findings also demonstrate that social media activities have been a source of participant recruitment. Potential participants (ie, middle-aged and older adults) learned about our research program through social media–advertised talks as well as social media post content, including our Research Awareness Series. This allowed us to increase the number of participant referrals to center-affiliated studies, supporting the promise of social media as a low-cost method of recruiting potential research participants [16,25,26]. Additional follow-up is needed to determine whether these participants have different demographics and retention characteristics than individuals recruited through in-person community outreach or other activities.

Our evidence suggests that innovative social media activities can also provide novel opportunities for the scientific community. For example, although our web-based *Memory Matters* talk series was primarily designed to share recent research findings with the lay community, it simultaneously provided professional development opportunities. Through this series, junior investigators receive mentorship on the principles and importance of science communication, specifically communicating science-related topics with the public [27,28]. Engaging staff and investigators in the development of social media content, such as writing accessible descriptions of their research programs and findings, can also provide science communication opportunities. This highlights an additional possible benefit of social media for centers or programs that may be considering whether to develop a social media presence or those exploring ways in which they might expand their programming.

Moreover, providing education and resources for adults in the local community, including individuals living with cognitive impairment and caregivers, is in alignment with international efforts promoting healthy aging, ADRD education and support, and dementia risk reduction [3,4]. While most of our educational content to date focused on these broad topics, future content could include materials that address ageism and ableism, and promote age-friendly [29] and dementia-friendly [30] activities and resources that ensure inclusive environments, empowerment, and sustained engagement with issues related to age and disease.

### Limitations

This study has limitations. First, this is a retrospective account of one center’s social media activities and their outcomes, and the number of individuals following the social media pages is still modest. Additional work is needed to understand the long-term impact of these activities on community education, center visibility, and clinical research recruitment, and to evaluate the types of content that lead individuals to express interest in participating in center-affiliated research studies. Second, we do not have data on the demographics of our social media followers to evaluate whether our content reached the target audience or the extent to which we are reaching individuals living with cognitive impairment. However, the recruitment metrics indicate that those expressing interest in participating in research were in the target age range. Social media and internet use is reportedly lower among the oldest-old, as well as individuals with cognitive impairment and poorer subjective health [31-36]; these groups may, therefore, be less likely to engage with these platforms. Third, we did not compare the efficacy of the 3 social media platforms described, although we believe that each allows us to reach unique audiences in different ways. Fourth, the individuals who have expressed interest in research have been predominantly female; additional efforts are needed to understand how to similarly reach males, as well as a greater proportion of individuals from diverse racial and ethnic backgrounds. Fifth, although our reach and impression rates are promising, our Facebook engagement rates are relatively low. Because this metric is an indicator of how, and the degree to which, our audience interacts with our content, these data suggest that much of our social media outreach may be unidirectional (ie, low levels of likes, comments, and content sharing). Future efforts should strive to improve this. Additionally, these measures were collected as monthly averages, which limited our ability to assess the impact of individual posts or specific content types. Furthermore, engagement data were not collected for Twitter. Finally, we describe the efforts of one Alzheimer’s Disease Research Center; the extent to which similar approaches generalize to other types of centers or programs remains to be determined.

Although social media may be an effective means for raising awareness about dementia, dispelling stigma, and highlighting positive aspects of clinical research [37], it is only one method within a wide range of strategies needed for engaging communities, individuals with cognitive impairment, and families on topics related to aging and ADRD [5,24]. Social media may supplement in-person community outreach, engagement, and partnerships, the latter of which have been reported to be important for building trust, particularly in minoritized communities [38-40]. It will furthermore be important to continually monitor use trends among target demographics, and evaluate the addition of, or migration to, other emerging platforms. We, nonetheless, hope these activities—or a subset thereof—may serve as an exemplar for other centers or programs, or provide ideas for new initiatives that can be implemented and improved upon.

### Conclusions

These data suggest that social media activities may have a measurable impact on the outreach, visibility, and recruitment activities of research centers, including National Institute on Aging–funded ADRCs. They also highlight the importance of tracking the success of outreach programs for evaluating outcomes. These data provide evidence of return on investment and support the continued use of social media for the above-mentioned purposes. Given the public’s use of social media as a source of health information [6], this may be an important means by which centers can present themselves as a reliable educational resource, educate the community, and share research findings with community stakeholders. Incorporating additional activities designed to test the efficacy of different approaches for attracting research participants is an important future direction.

## Abbreviations

ADRD: Alzheimer disease and related dementias
IRB: institutional review board
JH: Johns Hopkins
JHADRC: Johns Hopkins Alzheimer’s Disease Research Center
MACAB: Memory and Aging Community Advisory Board
ORE: Outreach, Recruitment, and Engagement
